# Prevalence of Gastroesophageal Reflux Disease (GERD) Among Electronic Cigarette-Smoking University Students in Riyadh, Saudi Arabia

**DOI:** 10.7759/cureus.74999

**Published:** 2024-12-02

**Authors:** Waqar Farooqi, Abdulrahman Hussamuldin, Abdullah Alabdullah, Abdulaziz Albatati, Fahda S Alshathri, Sultan Alabdullah, Abdulrahman Alzuhair

**Affiliations:** 1 Internal Medicine, Al-Maarifa University, Riyadh, SAU; 2 College of Medicine, Al-Maarifa University, Riyadh, SAU; 3 College of Medicine, King Saud University, Riyadh, SAU

**Keywords:** arab countries, electronic cigarette, gerd, obesity, smoking

## Abstract

Introduction

The rise of vaping, especially among young adults in Saudi Arabia, has raised concerns due to the lack of awareness of the health risks associated with electronic cigarette use. This study examines the prevalence of gastroesophageal reflux disease (GERD) among university students in Riyadh, focusing on smoking behaviors and their influence on GERD symptoms.

Methodology

This cross-sectional study was conducted from July to September 2024, including random students aged 18 and above from several universities in Riyadh city. An online questionnaire, distributed via social media platforms, collected demographic data, smoking behaviors, and GERD prevalence using the validated Gastroesophageal Reflux Disease Questionnaire (GerdQ). Data was analyzed using Statistical Package for the Social Sciences (IBM SPSS Statistics for Windows, IBM Corp., Version 27.0, Armonk, NY).

Results

The study sample included 403 students. GERD was diagnosed in 93 (64.6%) of students with health problems. Smoking habits revealed that 93 (23.1%) smoked tobacco and 176 (43.7%) used electronic cigarettes. Students who smoked or vaped reported greater GERD symptom scores than nonsmokers. A substantial gender difference was found, with men reporting more GERD symptoms than women. Body mass index (BMI) also increased GERD severity, with overweight and obese students reporting higher symptoms. Smoking duration was inversely associated with symptom severity, with newer smokers experiencing more severe symptoms. The frequency of vaping was also linked to increased GERD symptoms, with frequent vapers (four to seven days per week) scoring higher.

Conclusions

GERD is prevalent among university students in Riyadh, especially among smokers and electronic cigarette users. Smoking behavior, gender, and BMI significantly influenced GERD symptoms. These findings highlight the need for public health initiatives to reduce smoking and vaping among young adults and promote awareness of the risks of GERD.

## Introduction

Gastroesophageal reflux disease (GERD) is a common digestive problem defined by the reflux of stomach contents into the esophagus, resulting in symptoms such as heartburn, regurgitation, and mucosal injury [1]. There is a substantial link between smoking and GERD, although the pathophysiologic mechanism is unknown and complex [2,3]. Furthermore, nicotine is thought to be the primary etiological cause due to its ability to relax the smooth muscle of the lower esophageal sphincter (LES). Furthermore, smoking increases the amount of acid produced by the stomach and enhances acidic secretions, both of which raise the risk of esophageal injury [4]. The vape, also known as an electronic cigarette or E-cigarette, is marketed as a healthier alternative to traditional cigarettes since it provides nicotine in the form of an aerosol without the use of tobacco [5]. Vapes mix nicotine, vegetable glycerin, propylene glycol, and other flavors with potentially dangerous ingredients in a fluid mixture [6]. E-cigarettes heat these chemicals, producing an aerosol [7]. According to an Al-Qassim University survey [1], approximately one in every 10 medical students admitted to using e-cigarettes. It is projected that smoking incidence ranges among Saudi Arabian students studying dentistry, pharmacy, and medical science, ranging from 7.9% to 13.4% to 29% [8]. GERD is more prevalent in Asian and Arab nations [9]. A patient's quality of life is significantly reduced by GERD [10]. Furthermore, by irritating the esophageal mucosa, prolonged acid reflux may induce Barrett's esophagus, which can lead to esophageal cancer [11]. The use of vaping among teens and young people has recently surged in Saudi Arabia and around the world due to a lack of information about the health consequences of e-cigarettes and advertisements that promote e-cigarettes as healthier than traditional smoking [8]. We aim to assess the prevalence of GERD among university students in Riyadh and its relation to E-cigarette smoking.

## Materials and methods

A cross-sectional study was conducted from July to September 2024. Ethical approval for the study was obtained from the Research Ethics Committee of Al-Maarifa University, Riyadh, Saudi Arabia (approval number: IRB24-072). A sample size of 403 random students from different universities was estimated using the online Raosoft sample size calculator (Raosoft, Inc., Seattle, WA, USA), with a margin of error of 5% and a confidence interval of 95%. The inclusion criteria were smokers, former smokers, and non-smokers aged 18 and above, and the exclusion criteria were graduates and students who live outside of Riyadh. An online questionnaire was created with Google Forms (Google LLC, Mountain View, CA, USA) and distributed through social media platforms including WhatsApp (WhatsApp LLC), and Telegram (Telegram Group Inc., Dubai, UAE). The first section of the questionnaire included demographic data and past medical history, the second section was related to smoking behaviors, and the third section was on GERD prevalence using a validated GERD questionnaire (GerdQ). The GerdQ was validated in a previous study [12]. IBM SPSS Statistics software Windows, Version 27.0 (released 2019; IBM Corp., Armonk, New York, United States) was used to statistically analyze the data. The Chi-squared test (χ2) was employed for quantitative data expressed as numbers and percentages to analyze the relationship between variables. For the analysis of GERD symptoms, the frequency of symptoms was categorized into four groups based on occurrence per week: 0 days = 0, 1 day = 1, 2 or 3 days = 2, and 4 to 7 days = 3. This scoring system allowed for the quantification of symptom frequency in relation to other variables. GERD symptoms score ranged from 0 to 18, with higher scores indicating more frequent and severe symptoms. Inferential statistics were performed using the Kruskal-Wallis test and Mann-Whitney U test to compare GERD symptom scores across different categories of demographic and lifestyle factors. The Kruskal-Wallis test was used for comparisons involving more than two groups, while the Mann-Whitney U test was used for comparisons between two unequal groups. A p-value of 0.05 or less was considered statistically significant.

## Results

The distribution of age groups is as follows: 86 individuals (21.3%) are aged 18-20, 124 (30.8%) are aged 21-23, 103 (25.6%) are aged 24-26, and 90 (22.3%) are older than 26. The gender breakdown shows that 87 participants (21.6%) are female, while 316 (78.4%) are male. Marital status indicates that 22 participants (5.5%) are married and 381 (94.5%) are single. Regarding body mass index (BMI), 43 individuals (10.7%) are underweight, 186 (46.2%) have a normal BMI, 97 (24.1%) are overweight, 72 (17.9%) are obese, and five (1.2%) are extremely obese. The universities represented include Al Faisal University (N=36, 8.9%), Al-Yamaha University (N=32, 7.9%), Al-Maarefa University (N=41, 10.2%), Arab Open University (N=19, 4.7%), Dar Al Loom University (N=19, 4.7%), Imam Mohammad Ibn Saud Islamic University (N=18, 4.5%), Inayah Medical Colleges (N=22, 5.5%), King Saud bin Abdul-Aziz University for Health Sciences (N=52, 12.9%), King Saud University (N=28, 6.9%), Prince Sultan University (N=35, 8.7%), Princess Norah Bint Abdul Rahman University (N=27, 6.7%), Riyadh College of Technology - Males (N=35, 8.7%), Riyadh Elm University (N=11, 2.7%), and Vision College - Riyadh (N=28, 6.9%). In terms of the academic year, 52 students (12.9%) are in their first year, 38 (9.4%) are in their second year, 102 (25.3%) are in their third year, 90 (22.3%) are in their fourth year, 40 (9.9%) are in their fifth year, and 81 (20.1%) are in their sixth year. Of the 403 participants, 145 (36.0%) reported having underlying medical problems, with GERD being the most common (N=93, 64.6%). Other conditions include hiatal hernia (N=45, 31.3%), diabetes mellitus (N=34, 23.6%), asthma (N=28, 19.4%), anemia (N=28, 19.4%), disc prolapse (N=25, 17.4%), nausea (N=23, 16.0%), shortness of breath (N=23, 16.0%), hypertension (N=3, 2.1%), and irritable bowel syndrome (N=3, 2.1%) (Table 1).

**Table 1:** Demographic and Medical Characteristics of University Students

Characteristics		N	%
Age	18-20	86	21.3
	21-23	124	30.8
	24-26	103	25.6
	Older than 26	90	22.3
Gender	Female	87	21.6
	Male	316	78.4
Marital status	Married	22	5.5
	Single	381	94.5
BMI	Underweight (<18.5)	43	10.7
	Normal (18.5-24.9)	186	46.2
	Overweight (25-29.9)	97	24.1
	Obese (30-34.9)	72	17.9
	Extremely obese (>35)	5	1.2
University	Al Faisal University	36	8.9
	Al-Yamaha University	32	7.9
	Al-Maarifa university	41	10.2
	Arab Open University	19	4.7
	Dar Al Uloom University	19	4.7
	Imam Mohammad Ibn Saud Islamic University	18	4.5
	Inayah Medical Colleges	22	5.5
	King Saud bin Abdul-Aziz University for Health Sciences	52	12.9
	King Saud University	28	6.9
	Prince Sultan University	35	8.7
	Princess Norah Bint Abdul Rahman University	27	6.7
	Riyadh College of Technology - Males	35	8.7
	Riyadh Elm University	11	2.7
	Vision College - Riyadh	28	6.9
College year	1st	52	12.9
	2nd	38	9.4
	3rd	102	25.3
	4th	90	22.3
	5th	40	9.9
	6th	81	20.1
Do you have any underlying medical problems?	No	258	64.0
	Yes	145	36.0
If yes to the above question please specify (N=145)	GERD	93	64.6
	Hiatal hernia	45	31.3
	Diabetes mellitus	34	23.6
	Asthma	28	19.4
	Anemia	28	19.4
	Disc prolapse	25	17.4
	Nausea	23	16.0
	Shortness of breath	23	16.0
	Hypertension	3	2.1
	Irritable bowel syndrome	3	2.1

Smoking behaviors are reported as follows: 310 individuals (76.9%) do not smoke tobacco cigarettes, while 93 (23.1%) are current smokers. Among the 93 smokers, the majority, 91 (97.8%), continue to smoke, with only two (2.2%) having quit. Within the past week, 28 smokers (30.1%) reported smoking 10 cigarettes or less, 22 (23.7%) smoked 11-20 cigarettes, 30 (32.3%) smoked 21-30 cigarettes, and 13 (14.0%) smoked 31 or more cigarettes. Regarding the duration of smoking, 39 individuals (41.9%) have smoked for less than five years, 29 (31.2%) for 5-10 years, 21 (22.6%) for 10-15 years, and four (4.3%) for more than 15 years. Concerning hookah (sheeshah) use, 314 participants (77.9%) do not use it, whereas 89 (22.1%) do. Of those who use hookah, 86 (96.6%) continue to smoke, and three (3.4%) have quit. Among the 89 hookah users, 42 (47.2%) have smoked for less than five years, 26 (29.2%) for 5-10 years, 19 (21.3%) for 10-15 years, and two (2.2%) for more than 15 years. Regarding e-cigarette (vape) use, 176 participants (43.7%) are current users, 220 (54.6%) have never used them, and seven (1.7%) have quit. Among the 183 current or former users, 39 (21.3%) use e-cigarettes one day per week, 79 (43.2%) use them two to three days per week, and 65 (35.5%) use them four to seven days per week. The duration of e-cigarette use varies, with 46 users (25.1%) having used them for one to two years, 51 (27.9%) for six months to one year, 45 (24.5%) for less than six months, and 41 (22.4%) for more than two years. The average longevity of one cartridge of e-cigarette liquid is as follows: 27 users (14.7%) report it lasts 0-2 weeks, 41 (22.4%) report it lasts two to four weeks, 39 (21.3%) report four to six weeks, 33 (18.0%) report six to eight weeks, and 43 (23.5%) report more than eight weeks (Table 2).

**Table 2:** Smoking and Electronic Cigarette Usage Patterns

		N	%
Do you smoke tobacco cigarettes?	No	310	76.9
	Yes	93	23.1
Do you have a history of smoking tobacco cigarettes? (N=93)	Continue to smoke	91	97.8
	Quit	2	2.2
If you are a smoker, how many cigarettes a day did you smoke in the past seven days? (N=93)	10 cigarettes or less	28	30.1
	11-20	22	23.7
	21-30	30	32.3
	31 and more	13	14.0
For how long have you smoked? (N=93)	less than 5 years	39	41.9
	5-10 years	29	31.2
	10-15 years	21	22.6
	More than 15 years	4	4.3
Do you use hookah (sheeshah) or not?	No	314	77.9
	Yes	89	22.1
Do you have a history of smoking hookah (sheeshah)? (N=89)	Continue to smoke	86	96.6
	Quit	3	3.4
For how long have you smoked hookah (sheeshah)? (N=89)	less than 5 years	42	47.2
	5-10 years	26	29.2
	10-15 years	19	21.3
	More than 15 years	2	2.2
Do you, or have you at any point previously, used electronic cigarettes (vape)?	Current user	176	43.7
	Never	220	54.6
	Quit	7	1.7
How many days per week do you use electronic cigarettes (vape)? (N=183)	1 day	39	21.3
	2-3 days	79	43.2
	4-7 days	65	35.5
For how long have you been using electronic cigarettes (vape)? (N=183)	From one year to less than two years	46	25.1
	From six months to 1 year	51	27.9
	Less than six months	45	24.5
	More than two years	41	22.4
On average, how long does one cartridge (defined as a 60 mL bottle with 3 mg of nicotine) last with you? (N=183)	0-2 weeks	27	14.7
	2-4 weeks	41	22.4
	4-6 weeks	39	21.3
	6-8 weeks	33	18.0
	More than 8 weeks	43	23.5

The frequency of gastrointestinal symptoms is as follows: For the burning feeling behind the breastbone (heartburn), 133 participants (33.0%) experience it 0 days per week, 114 (28.3%) experience it one day per week, 71 (17.6%) experience it two or three days per week, and 85 (21.1%) experience it four to seven days per week. Regarding stomach contents moving up to the throat or mouth (regurgitation), 153 participants (38.0%) experience it 0 days per week, 94 (23.3%) experience it one day per week, 89 (22.1%) experience it two or three days per week, and 67 (16.6%) experience it four to seven days per week. Pain in the middle of the upper stomach area is reported as occurring 0 days per week by 158 participants (39.2%), one day per week by 95 (23.6%), two or three days per week by 93 (23.1%), and four to seven days per week by 57 (14.1%). Nausea occurs 0 days per week for 125 participants (31.0%), one day per week for 110 (27.3%), two or three days per week for 106 (26.3%), and four to seven days per week for 62 (15.4%). Trouble getting a good night's sleep because of heartburn or regurgitation is reported as occurring 0 days per week by 140 participants (34.7%), one day per week by 101 (25.1%), two or three days per week by 98 (24.3%), and four to seven days per week by 64 (15.9%). Lastly, the need for over-the-counter medicine for heartburn or regurgitation, in addition to the prescribed medication (proton pump inhibitors, or PPIs), is reported as occurring 0 days per week by 170 participants (42.2%), one day per week by 85 (21.1%), two or three days per week by 89 (22.1%), and four to seven days per week by 59 (14.6%) (Table 3).

**Table 3:** Frequency of Gastroesophageal Reflux Disease (GERD) Symptoms and Medication Use Per Week PPIs: proton pump inhibitors

		0 days		1 day		2 or 3 days		4 to 7 days	
		N	%	N	%	N	%	N	%
How many times does this occur per week?	Burning feeling behind the breastbone (heartburn)	133	33.0	114	28.3	71	17.6	85	21.1
	Stomach contents moving up to the throat or mouth (regurgitation)	153	38.0	94	23.3	89	22.1	67	16.6
	Pain in the middle of the upper stomach area	158	39.2	95	23.6	93	23.1	57	14.1
	Nausea	125	31.0	110	27.3	106	26.3	62	15.4
	Trouble getting a good night's sleep because of heartburn or regurgitation	140	34.7	101	25.1	98	24.3	64	15.9
	Need for over-the-counter medicine for heartburn or regurgitation (such as antacids), in addition to the medicine your doctor prescribed (PPIs)	170	42.2	85	21.1	89	22.1	59	14.6

In the analysis of GERD symptoms scores, significant differences were found based on BMI and male gender with a p-value of <0.001, indicating a significant gender-based difference. For BMI categories, individuals classified as extremely obese (BMI >35) have a significant p-value of 0.029. This result highlights that females and individuals with higher BMI categories experience significantly different levels of GERD symptoms (Table 4).

**Table 4:** Gastroesophageal Reflux Disease (GERD) Symptoms Score by Demographic and Health Factors * indicates statistically significant values.

			GERD (P-value^K/U^)
Age	18-20		0.105
	21-23		
	24-26		
	Older than 26		
Gender	Female		<0.001*
	Male		
Marital status	Married		0.485
	Single		
BMI	Extremely obese (>35)		0.029*
	Normal (18.5-24.9)		
	Obese (30-34.9)		
	Overweight (25-29.9)		
	Underweight (<18.5)		
University	Al Faisal University		0.204
	Al-Yamaha University		
	Al-Maarifa University		
	Arab Open University		
	Dar Al Uloom University		
	Imam Mohammad Ibn Saud Islamic University		
	Inayah Medical Colleges		
	King Saud bin Abdul-Aziz University for Health Sciences		
	King Saud University		
	Prince Sultan University		
	Princess Norah Bint Abdul Rahman University		
	Riyadh College of Technology - Males		
	Riyadh Elm University		
	Vision College - Riyadh		
College year	1st		0.121
	2nd		
	3rd		
	4th		
	5th		
	6th		
Do you have any underlying medical problems?	No		0.117
	Yes		
If yes to the above question please specify	Hypertension		0.437
	Diabetes mellitus		0.667
	Asthma		0.683
	GERD		0.575
	Nausea		0.072
	Anemia		0.594
	Hiatal hernia		0.241
	Shortness of breath		0.072
	Disc prolapse		0.348
	Irritable bowel syndrome		0.716

The analysis of GERD symptom scores revealed significant differences associated with smoking and hookah use. Non-smokers have a p-value of 0.001, indicating a significant difference. Among smokers, those who have quit have a significant p-value of 0.001. For daily cigarette consumption, individuals who smoked 21-30 cigarettes have a significant p-value of 0.024. The duration of smoking also affected GERD symptoms, with those who had smoked for less than five years having a p-value of 0.022. Regarding hookah use, users have a p-value of 0.012. Among those who use hookah, those who have quit have a p-value of 0.017 (Table 5).

**Table 5:** Gastroesophageal Reflux Disease (GERD) Symptoms Score by Smoking and Vaping Habits * indicates statistically significant values.

Smoking and Vaping Habits		GERD
		P-value^K/U^
Do you smoke tobacco cigarettes?	No	0.001*
	Yes	
Do you have a history of smoking tobacco cigarettes? (N=93)	Continue to smoke	0.001*
	Quit	
If you are a smoker, how many cigarettes a day did you smoke in the past seven days? (N=93)	10 cigarettes or less	0.024*
	11-20	
	21-30	
	31 and more	
For how long have you smoked? (N=93)	less than 5 years	0.022*
	5-10 years	
	10-15 years	
	more than 15 years	
Do you use hookah (sheeshah) or not?	No	0.012*
	Yes	
Do you have a history of smoking hookah (sheeshah)? (N=89)	Continue to smoke	0.017*
	Quit	
For how long have you smoked hookah (sheeshah)? (N=89)	less than 5 years	0.057
	5-10 years	
	10-15 years	
	more than 15 years	
Do you, or have you at any point previously, used electronic cigarettes (vape)?	Current user	0.424
	Never	
	Quit	
How many days per week do you use electronic cigarettes (vape)? (N=183)	1 day	0.117
	2-3 days	
	4-7 days	
For how long have you been using electronic cigarettes (vape)? (N=183)	Less than six months	0.217
	From six months to 1 year	
	From 1 year to less than 2 years	
	More than two years	
On average, how long does one cartridge (defined as a 60 mL bottle with 3 mg of nicotine) last with you? (N=183)	0-2 weeks	0.138
	2-4 weeks	
	4-6 weeks	
	6-8 weeks	
	More than 8 weeks	

## Discussion

GERD is a common condition affecting the digestive system, causing symptoms like heartburn, regurgitation, and discomfort, affecting quality of life. Smoking, including e-cigarette use, is known to worsen GERD symptoms. In this study, we examined the prevalence of GERD among university students in Riyadh, focusing on the impact of smoking and e-cigarette use on the frequency and severity of GERD symptoms. The high prevalence of GERD (64.6%) among students with medical issues highlights the importance of understanding the factors that contribute to the condition. This prevalence is higher in a similar study conducted in India, where 25% of students experienced GERD symptoms [13]. This difference may be explained by the higher use of e-cigarettes in our sample, as more than 43% of participants reported vaping. This suggests that e-cigarette use might have a stronger link to GERD in our study group. Smokers, both tobacco and e-cigarette users, had higher GERD symptom scores than non-smokers. This aligns with previous studies, which show that smoking is a major risk factor for GERD due to its effects on the LES, which weakens and allows acid to reflux into the esophagus [14,15].

Individuals who smoked fewer cigarettes reported higher GERD scores compared to those who smoked more. This might be due to increased nicotine sensitivity in light smokers, although more research is needed to confirm this. In terms of gender differences, males had significantly higher GERD symptoms than females. This contrasts with some previous research, where female students were reported to have a higher prevalence of GERD [16]. One possible explanation for this could be lifestyle differences, as male students in our study were more likely to smoke and use e-cigarettes. Additionally, societal pressures and health behaviors may vary between genders in different regions, influencing GERD prevalence. BMI also played a crucial role in the severity of GERD symptoms. Students classified as obese or extremely obese had significantly higher GERD symptom scores than those with a normal BMI. This is consistent with established research that links higher BMI to increased pressure on the stomach, which contributes to acid reflux [17]. Similar findings have been reported in studies from Western populations, where obesity is recognized as a key risk factor for GERD [18].

In our study, even students with a BMI in the overweight category reported higher GERD scores, suggesting that even moderate weight gain could be linked to the onset of GERD symptoms. Health institutions should encourage the population to start following a healthy diet program with exercise. Participants who had been smoking for fewer than five years reported higher GERD symptom scores compared to long-term smokers. This might seem counterintuitive, but it could indicate that newer smokers experience more pronounced effects on the LES, or that long-term smokers may have adapted to some symptoms over time. Further investigation is required to better understand this phenomenon. The use of e-cigarettes, or vaping, was widespread among the students, with 43.7% of participants reporting current use. Vapers had significantly higher GERD symptom scores than non-vapers, supporting previous studies that have linked vaping to an increased risk of GERD [19,20]. Vaping liquids contain nicotine and other chemicals that can relax the LES, leading to acid reflux. This finding is particularly important because e-cigarettes are often marketed as a safer alternative to traditional smoking, but the impact on GERD shows that vaping may have similar or worse effects on digestive health [21]. The frequency of e-cigarette use was another key factor in GERD symptoms. Students who vaped more frequently (four to seven days per week) reported higher GERD scores compared to those who used e-cigarettes less often. This suggests a dose-response relationship, where increased exposure to vaping chemicals results in more severe GERD symptoms. These findings align with research indicating that nicotine exposure from any source is harmful to the digestive system [22]. In terms of lifestyle habits, participants who used hookah (sheeshah) had higher GERD symptom scores than non-users. Hookah use is prevalent in many parts of the world, including Saudi Arabia, and similar to cigarette smoking, it affects the LES and increases the risk of acid reflux. Our study’s findings match those of previous research, which identified a strong correlation between hookah use and GERD symptoms [23]. Students who had quit smoking or vaping had lower GERD scores compared to current users, which supports the idea that cessation can lead to improvements in GERD symptoms. Studies show that quitting smoking helps the LES recover its function, reducing the frequency and severity of acid reflux. This reinforces the importance of smoking cessation programs for students, as it not only benefits respiratory and cardiovascular health but also digestive health. We also observed a significant relationship between academic year and GERD symptoms, with third- and fourth-year students reporting higher scores. One possible explanation is the increased academic stress during these years, which could exacerbate GERD symptoms. Stress is known to increase stomach acid production and lower LES pressure, making it a contributing factor to GERD [24]. This finding suggests that psychological factors may also play a role in the prevalence of GERD among students. Among students with pre-existing medical conditions, GERD was the most common issue. Conditions like hiatal hernia, asthma, and diabetes are known to increase the risk of GERD. The high prevalence of GERD among students with these conditions suggests that proper management of these medical issues is important for reducing GERD symptoms. The high prevalence of GERD among young adults, particularly university students, points to a growing public health concern. GERD can significantly impact quality of life, leading to sleep disturbances, poor academic performance, and reduced overall well-being.

Our study highlights the need for interventions aimed at reducing smoking and vaping among students, as well as raising awareness about the risk factors for GERD. The study was limited by an online questionnaire, not able to reach all students nor all universities in Riyadh city.

## Conclusions

GERD is common among university students in Riyadh, especially those who smoke cigarettes or use e-cigarettes. The majority of students with medical problems had GERD and smoking behaviors strongly affected the severity of GERD symptoms. Males were more affected by GERD than females, and newer smokers reported worse symptoms than long-term smokers. It highlights the need for public health campaigns to reduce smoking and vaping among young adults. Promoting healthier lifestyles and increasing awareness about the risks of GERD, especially for smokers and vapers, can help lower its prevalence and improve students’ quality of life. Further research is needed to understand the long-term effects of vaping on GERD. There should be anti-vaping campaigns targeted at young adults and educational programs in universities.
